# Trends and Future Projections in Ultrasonic Welding Research for Hybrid Materials

**DOI:** 10.3390/polym17081124

**Published:** 2025-04-21

**Authors:** Jedaías J. Silva, Rafael G. C. da Silva, Carolina L. Morelli, Edwar A. T. López, Tiago F. A. Santos

**Affiliations:** 1 Brazilian Institute for Material Joining and Coating Technologies—INTM, Federal University of Pernambuco, Recife 50740-540, Brazil; jedaias.januario@ufpe.br (J.J.S.); carolina.morelli@ufpe.br (C.L.M.); 2 Department of Chemical Engineering, Federal University of Pernambuco, Recife 50740-590, Brazil; rafael.casanova@ufpe.br; 3 Department of Mechanical Engineering, Federal University of Pernambuco, Recife 50740-550, Brazil; 4 Department of Mechanical Engineering, Research Group GEA, University of Antioquia, Medellín 050010, Colombia; eandres.torres@udea.edu.co

**Keywords:** ultrasonic welding, reinforced polymer, aluminum, composite, joining, Scopus, Web of Science

## Abstract

Ultrasonic welding has gained interest from various researchers and industries worldwide, particularly for joining dissimilar materials in sectors such as aerospace, aeronautics, and electronics. This paper presents a comprehensive bibliometric review aimed at mapping the evolving landscape of ultrasonic welding research. Through the systematic analysis of 1913 scientific documents, it identifies key advances, challenges, and future directions in the field. Furthermore, the bibliometric analysis sheds light on annual scientific production, prolific authors and institutions, scientific contribution per country, and methodological approaches. The global collaboration network comprises countries from all continents, with a prominent presence in Europe, Asia, and the Americas and less representation from African and Oceanian countries. China and the United States dominate the field in terms of scientific document production, international collaborations, and citations, with Germany also standing out for leading the number of citations in research related to hybrid metal/polymer joining. This review aims to serve as a valuable resource for researchers, practitioners, and policymakers interested in the advancements and future directions of ultrasonic welding for hybrid materials.

## 1. Introduction

Ultrasonic welding has established itself as a promising technique across various industries, including the aerospace [1,2,3,4], electronics [5,6,7], and automotive [8,9] sectors, particularly for joining dissimilar materials such as metals and polymers. The technique consists of using high-frequency ultrasonic vibrations to join materials without the need for adhesives, bolts, or soldering materials. Mechanical vibrations at ultrasonic frequencies (typically 20 kHz to 70 kHz) generate heat through friction, causing the materials to fuse at their interface. This ultrasonic welding process provides high precision in force application, resulting in stronger joints with reduced distortion [4,10,11].

In recent years, the use of sustainable technologies that provide greater safety and cost reduction has become increasingly necessary. The technological demands of the aerospace and automotive industries are driven by three critical factors: weight, performance, and durability [12,13]. Weight reduction is directly linked to decreased fuel consumption, lower emissions of harmful gasses, reduced noise pollution, increased payload capacity, and even lower mission costs [9,14,15]. The development of multifunctional materials or manufacturing methods capable of simultaneously providing mass reduction, significant mechanical strength, and radiation protection represents an alternative approach to enhancing system performance. The durability of an aircraft, on the other hand, is directly tied to mission safety, crew security, and the costs associated with equipment maintenance.

It is estimated that every hour that an aircraft remains grounded due to the premature failure of a critical component incurs a cost of USD 10,000 for the airline [12], representing a significant maintenance expense for a company already burdened with high operational costs. Therefore, to meet the demands of these sectors, a greater quantity of lightweight and high-strength metals, such as aluminum, titanium, or magnesium alloys, or fiber-reinforced polymer composites, is required for engineering structures [16].

Lightweight and cost-effective designs, replacing monolithic components with multimaterial parts, are a promising approach to achieving this goal [17]. However, to design with composite materials, appropriate joining methods are required [16]. Ultrasonic welding (USW) for the formation of hybrid joints has gained attention for addressing the recent needs of the aerospace sector [9,18]. This technique is suitable for plastics, soft non-ferrous metals such as copper alloys, aluminum, brass, gold, and silver [19], composites, and dissimilar joints [16,20]. The ultrasonic technique is capable of producing welded joints between metals that remain in the solid state and plastic parts that undergo localized fusion.

Feistauer et al. [19] developed hybrid joints by the USW of a titanium and aluminum alloy (Ti-6Al-4V) with glass fiber-reinforced polyetherimide (GF-PEI) using the u-joining method. The authors applied Box–Behnken experimental design for the optimization of parameters and the production of stronger joints. The study concluded that, for welding energy values of 2012.1 J, a welding pressure of 14.7 psi, and an oscillation amplitude of 52 μm, the maximum shear strength (3608 ± 417 N) and lowest lack of penetration (0.10 ± 0.05 mm) were achieved.

Lionetto, Balle, and Maffezzoli [21] studied the USW process to join aluminum alloy (AA5754) sheets to carbon fiber-reinforced epoxy resin composites, introducing a polyamide 6 (PA6) film to overcome the limitations of thermoset resins at high temperatures. The optimization of welding energy and force resulted in an average adhesion of 34.8 MPa. Morphological analysis revealed direct contact or the insertion of carbon fibers into the aluminum, with the interface showing pores and cracks due to intense plastic deformation.

For many years, aluminum alloys have been used as structural materials [22,23,24]. Among the applications of ultrasonic welding in aluminum alloys, the following stand out: the manufacturing of fuselage panels [25], wings, and internal finishing components of aircraft [26], as well as the production of connectors and battery enclosures in automotive applications [27]. Therefore, USW is a modern and essential technology that drives the use of aluminum alloys in innovative applications, aligning with trends of lightweight and sustainable manufacturing in today’s industries [28].

Engineering polymers reinforced with glass fibers or carbon fibers have gained prominence in ultrasonic welding processes due to their superior mechanical properties, such as high tensile strength and elastic modulus, combined with lightness and chemical resistance [29,30,31]. In the ultrasonic welding process, fiber reinforcement presents additional challenges, such as the non-uniform distribution of heat and the risk of the thermal degradation of the polymer matrix. However, the use of optimized parameters, such as amplitude, energy, power, welding pressure, force, and welding time, enables the creation of robust and durable interfaces [32]. Furthermore, the presence of fibers enhances mechanical anchoring and contributes to a high-performance interface, especially when combined with metallic materials in hybrid joints, expanding the potential application of these composites in lightweight and advanced structures.

Scientometric analysis is a powerful tool utilized in this paper to visualize the scientific growth of ultrasonic welding and its impact. Through these analyses, it is possible to identify the most influential authors, main journals, publication trends, emerging topics, and collaboration networks. This work provides a detailed spectrum of the topic’s evolution, offering relevant information for researchers, practitioners, and policymakers interested in the advancements and future directions of ultrasonic welding for hybrid materials.

In this context, this study conducted a scientific mapping of the process of producing hybrid joints through ultrasonic welding, with an emphasis on aluminum alloys and reinforced engineering polymers, which are widely used in the aforementioned sectors, aiming to demonstrate the relevance of the topic and opportunities for future research.

## 2. Materials and Methods

### 2.1. Databases Selection

The selection of databases for the search was based on the amount of available content and their relevance to the field of materials engineering and science. The Scopus and Web of Science (WoS) databases were used, both widely recognized for their coverage, quality of indexed records, high reliability, and broad adoption within the global community. These databases also offer complementary tools that enable the panoramic visualization of the research over time, by author, and by topic.

### 2.2. Search Strategy

The research was conducted through the selection of keywords that effectively encompassed relevant works on the topic. The search queries were applied to the selected databases, as shown in Table 1, to filter the documents based on the fields of title, abstract, and keywords chosen by the authors.

The use of Boolean operators and wildcard characters (*) ensured greater comprehensiveness in retrieving documents, preventing the loss of relevant information due to differences in terminology used by the authors. This strategic approach enabled efficient data collection, providing a solid foundation for subsequent analyses.

### 2.3. Data Processing

For the removal of duplicate files and the processing of the data obtained from the search, the LibreOffice Calc spreadsheet editor (version 24.8.3.2), an R script running in the open-source RStudio program (version 2024.09.0+375), and the Bibliometrix package in the R environment were used. These software tools facilitated the organization and processing of the obtained data, as well as the removal of duplicate documents from the two different databases. The use of RStudio was motivated by its accessibility, user-friendly interface, robustness of the available libraries for data processing, and widespread use in the academic community. Bibliometrix provides advanced features for conducting bibliometric and scientometric analyses, such as the visualization of collaboration networks, co-occurrence, keywords, and the analysis of temporal trends, making data interpretation more interactive.

Data collection was conducted between 14 and 18 October 2024, ensuring that the research captured the most recent publications. Only scientific articles within the scope of the research were considered, aiming to obtain results that align with current trends in scientific innovation. Therefore, other types of documents, such as reviews, conference papers, and book chapters, were excluded to ensure greater consistency of the data.

## 3. Results

**Figure 1:**
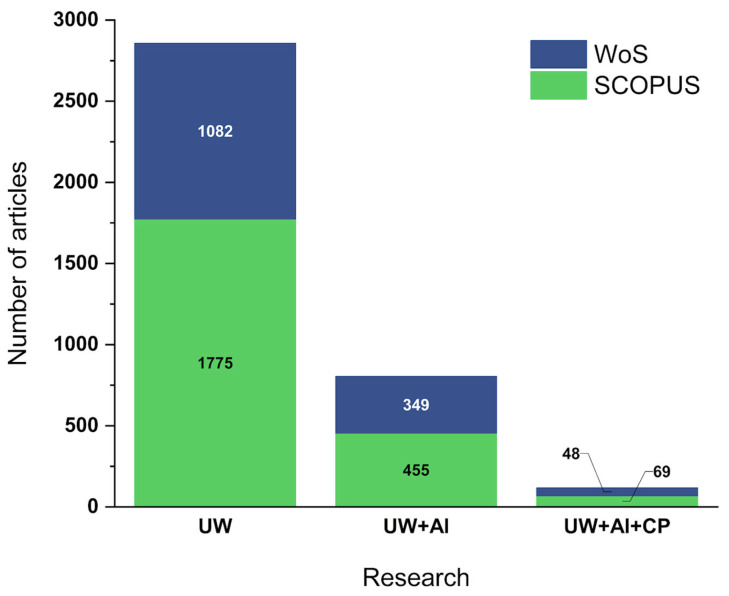
Number of articles found by search and by database.

The number of articles found through the database search is represented in Figure 1. Most of the articles were found in the Scopus database for the three searches conducted, likely due to the larger number of indexed journals (~43,400 journals). The term “Ultrasonic Welding” (UW) resulted in the highest number of results—being a broader topic—with 1775 articles in Scopus and 1082 in WoS. After data processing, 1473 duplicate articles were found. By adding the term “Aluminum” (UW + AL) to the search, the number of articles decreased to 445 and 349 in Scopus and WoS, respectively, with 350 of these articles being duplicates. Adding the terms “Composite” and “Polymer” (UW + AL + CP) further reduced the academic output, returning 69 articles in Scopus and 48 in WoS, with 42 duplicate documents.

In total, after the processing and removal of duplicates, 1913 articles were used. Of these, 1384 were obtained through the search for “Ultrasonic Welding”, 454 from the search for “Ultrasonic Welding + Aluminum”, and 75 articles from the search for “Ultrasonic Welding + Aluminum + Composite OR Polymer”. These articles were used for the preparation of the analyses. In addition to the search conducted in scientific articles, another analysis was performed on review articles. Detailed data can be found in the Supplementary Materials.

### 3.1. Ultrasonic Welding (UW)

**Figure 2:**
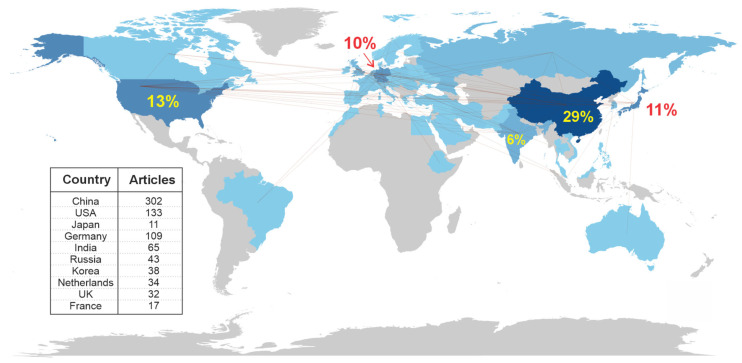
Network of collaboration of countries with publications on ultrasonic hybrid welding.

Figure 2 provides a detailed illustration of the global distribution of the 1384 articles found by country from 1979 to 2024. The map highlights, in shades of blue, the contribution of each country to the topic in question. The darker the blue tone, the greater the country’s contribution to the topic, while regions in gray indicate a near absence of articles. The legend shows the ten countries with the highest number of articles. China has the largest representation with 29% of the articles, followed by the USA (13%), Japan (11%), Germany (10%), and India (10%). The map also shows lines representing international collaborations among the authors.

The global collaboration network consists of countries from various continents, including Europe, Asia, the Americas, and even some countries from Africa and Oceania. China stands out as the country with the highest number of collaborations, with 28 interactions with the USA, as well as several collaborations with countries such as the United Kingdom, Japan, and Sweden. The USA is also well represented, with 28 collaborations with China, in addition to interactions with countries such as Japan, Germany, South Korea, and Israel. Countries like Germany, France, Japan, and India have a strong presence, indicating significant research activity related to ultrasonic welding. These patterns may indicate specific research areas or centers of excellence in ultrasonic welding. Some countries are beginning to stand out in collaborations, such as Indonesia, Morocco, and Cyprus, which appear with a few lower-frequency collaborations.

This network reflects the exchange of knowledge and technology related to ultrasonic welding, with a clear predominance of collaboration between industrialized nations and those innovating in materials and manufacturing. Brazil, for example, has some collaborations with countries like Austria and Germany, but its participation in collaborations is more modest compared to the countries mentioned above.

**Figure 3:**
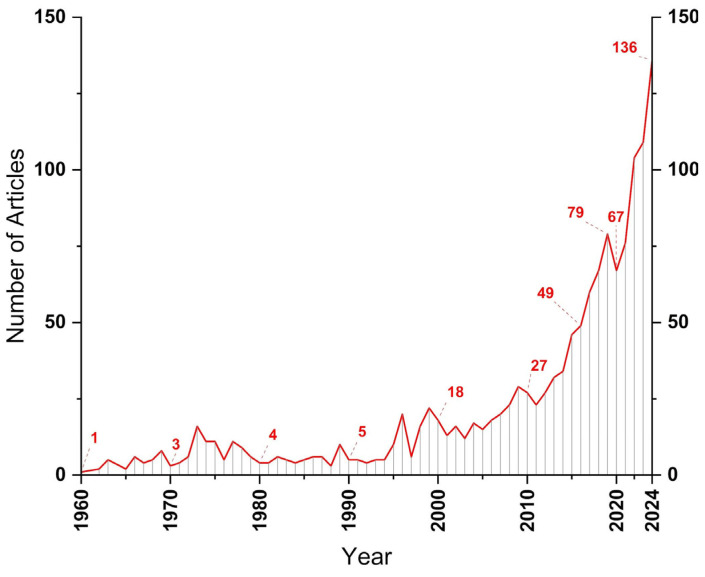
Temporal evolution of articles from the UW search.

Figure 3 presents the temporal distribution of the articles. A significant increase in the number of publications is noticeable, especially in the last few decades. In the mid-1990s, a subtle growth in the number of publications was observed; 1996 was marked by the production of 20 articles.

In the 2010s, there was a considerable growth rate in interest in the topic, rising from 27 articles in 2010 to 79 in 2019, likely due to the increased use of carbon fiber-reinforced polymers (CFRPs) and glass fiber-reinforced polymers (GFRPs) in the automotive, aerospace, and energy industries. The adoption of ultrasonic welding in these areas proved efficient due to its ability to join these materials effectively without compromising their properties or damaging the integrity of the equipment. The electronics industry benefited greatly from ultrasonic welding, becoming essential in the manufacturing of electronic devices and connectors.

In the context of mobile and automotive devices, ultrasonic welding is used in the assembly of lithium-ion batteries, commonly found in smartphones and hybrid or electric cars, as well as chargers and other essential components.

At the same time, the growing demand for new joining techniques with advanced materials has driven the development of solutions aimed at reducing weight and improving energy efficiency, particularly in the transportation and electronics industries.

In this context, technological advancements, such as more advanced welding systems with greater precision in controlling parameters like amplitude, frequency, force, and time, enabled more reliable results with higher reproducibility, attracting the interest of researchers and industries. Furthermore, the integration of ultrasonic welding systems into automated assembly lines increased productivity and reduced costs, consolidating large-scale applications.

There was a decline in the number of publications in 2020, with approximately 67 articles published. Coincidentally, this year marked the peak of the COVID-19 pandemic, but it still represented a significant number of publications, highlighting the relevance of the field even during times of crisis.

**Figure 4:**
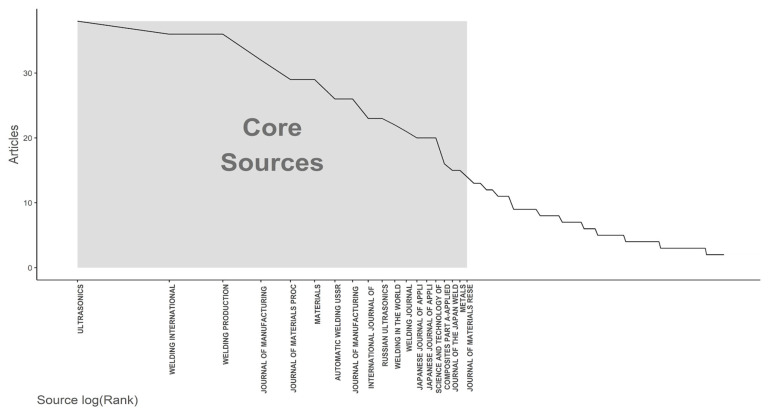
Bradford’s law chart for ultrasonic welding.

The Bradford’s law chart, Figure 4, highlights the distribution of articles across academic journals [33,34,35]. Bradford’s principle states that a small number of journals concentrate most of the relevant articles on a given topic, while the remaining journals contain fewer relevant articles, distributed exponentially across three zones. Zone 1 (core sources) is characterized by a few journals with a high number of articles, Zone 2 includes more journals but with fewer articles each, and finally, Zone 3 consists of a large number of journals, each containing only a few articles. The journals included in the core zone total 19 and are the ones that publish the most scientific articles, while the remaining journals outside this region total 449. The five journals with the highest number of publications are Ultrasonics (38), Welding International (36), and Welding Production (36), all with the same number of articles. The number of articles decreases as the graph moves to the right, with the last journal in the core zone being the Journal of Materials Research and Technology (JMR&T), which has 14 published articles—still a significant number—demonstrating a high concentration of articles in a relatively small number of journals.

The journals cover a variety of topics within the fields of welding, manufacturing processes, materials, and technological applications. Some, such as Welding International, Welding Production, Welding Journal, and Metals, primarily focus on metal welding, while there is still a limited representation of journals specifically dedicated to polymers, highlighting a field with gaps for the development and dissemination of new research.

An analysis was conducted on the journals listed in the core sources zone of the Bradford graph, incorporating the quartile classification of each journal, as shown in Table 2. The quartiles were obtained from the main journal ranking systems used by Scopus and WoS, namely the Journal Citation Reports (JCR) by Clarivate and the SCImago Journal Rank (SJR).

High-impact journals (Q1) in the scientific community included the Journal of Manufacturing Processes, Journal of Materials Processing Technology, Science and Technology of Welding and Joining, Composites Part A—Applied Science and Manufacturing, and the Journal of Materials Research and Technology, accounting for a total of 195 articles.

Journals with moderate impact (Q2) included Materials, International Journal of Advanced Manufacturing Technology, Welding in the World, Welding Journal, Japanese Journal of Applied Physics, Japanese Journal of Applied Physics Part 1, and Metals, totaling 150 articles.

The only journal classified in the fourth quartile (Q4), indicating a lower impact, was Welding International, with only 36 articles. The remaining journals had no available quartile information and accounted for 100 articles.

These results indicate that a significant portion of the articles were published in high-impact (Q1) and moderate-impact (Q2) journals, suggesting a high level of confidence in the sources used. Although the absence of approximately 21% of the quartile data limits a comprehensive evaluation, the overall distribution suggests a predominance of publications in highly relevant journals in the field.

**Figure 5:**
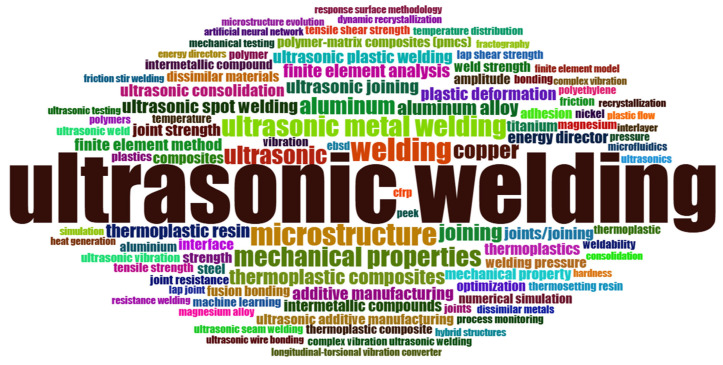
Word cloud.

Figure 5 presents a word cloud of the 100 most frequently used keywords by authors in the articles. At the center, the keyword “ultrasonic welding” appears most frequently, mentioned 517 times, followed by “welding” with 57 occurrences, and then “microstructure”, “ultrasonic metal welding”, and “mechanical properties” with 54, 47, and 42 mentions, respectively. The terms “aluminum”, “copper”, “titanium”, and “magnesium” show the diversity of the metallic materials addressed in the research, which are used across industries such as aerospace, automotive, and electronics. Additionally, the terms “thermoplastic composites” and “polymer-matrix composites (PCMS)” reveal the versatility of the technique, which can be applied to both metals and reinforced polymers. Furthermore, the terms “finite element analysis”, “simulation”, “response surface methodology”, and “machine learning” suggest a trend of integration with computational methods and advanced statistical analyses, aimed at improving process efficiency and precision while meeting the needs of high-performance industrial applications.

**Figure 6:**
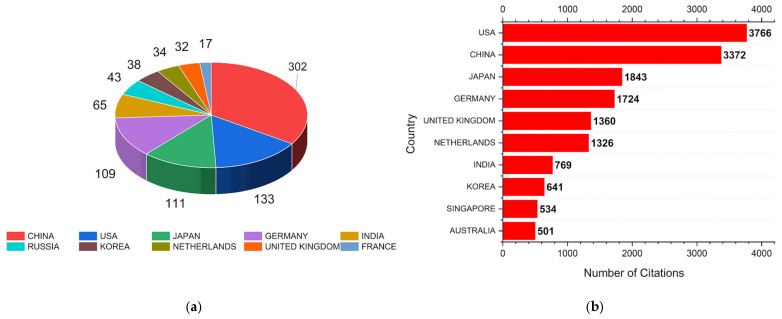
The graphs represent (**a**) the number of articles produced by country and (**b**) the number of citations obtained by country in the UW search.

It is possible to verify in Figure 6a, which shows the article production by country, that China stands out with 302 articles, which is more than double the number of articles from the United States (133), which ranked second. Japan (111) and Germany (109) are in third and fourth place, respectively, with a very small difference in the number of articles. Similarly to citations, Figure 6b shows that the Asian and European countries lead in the number of publications. The United States and China dominate in citation numbers, with 3766 and 3372, respectively. This indicates global leadership in scientific production. The data indicate a predominance of Asian and European countries, with only the United States representing America. Asia has 7159 citations and is represented by Japan, India, South Korea, and Singapore. Europe, with 4410 citations, is represented by Germany, the United Kingdom, and the Netherlands. This indicates that the topic is highly concentrated in regions of high industrial performance. Although India is an emerging country, it has gained prominence in this field, signaling industrial and scientific growth in the sector.

The United States leads as the only country from the Americas and has the highest number of citations per article. Its articles are more frequently cited than those of China, which ranks second in production, showing a higher impact per article. Countries with high citations per article include the United Kingdom (32 articles and 1360 citations) and the Netherlands (34 articles and 1326 citations), indicating significant influence in the field due to being highly referenced. South Korea, Japan, and Germany show relatively good impact despite having fewer articles.

Table 3 presents the articles with the highest number of citations. Wool et al. [36] published the article “Welding of Polymer Interfaces” with 359 citations. This article provides a detailed analysis of the factors influencing the mechanical properties of welded polymer joints. It highlights the welding mechanisms, such as surface rearrangement, wetting, diffusion, and randomization, which are essential for developing resistance at the polymer interface. Furthermore, the article explains the microscopic deformation mechanism, including the disentanglement of polymer chains and the rupture of bonds, which affect the durability of the weld strength. There is also a strong emphasis on the relationship between the interface structure and mechanical performance, noting that effective interdiffusion enhances strength, while the opposite results in brittleness. The article also discusses the complex diffusion patterns formed by polymer chains through fractal geometry, concluding that this is an important aspect of welding that has a direct effect on the interfacial structure and mechanical properties. However, more accurate molecular dynamics models are needed to better describe the complexity of this process.

Balle, Wagner, and Eifler [37] published the article titled “Ultrasonic Metal Welding of Aluminium Sheets to Carbon Fibre Reinforced Thermoplastic Composites”, which has 219 citations. The authors performed dissimilar ultrasonic welds between AA5754 sheets and PA66 composites reinforced with carbon fiber. A central composite design was used to optimize the welding parameters, which included welding force, oscillation amplitude, and welding energy. The study showed that an oscillation amplitude of 40.5 μm improved the contact between the metal sheet and the fibers, increasing the shear tensile strength of the joints. Amplitudes greater than this value caused damage to the fiber arrangement and the polymer, decreasing the strength. Therefore, a welding amplitude of 40 μm was maintained, and the maximum shear tensile strength obtained was 31.5 MPa, for a welding energy of 2160 W and a constant force of 140 N. The welding time was 3.5 s, and the maximum temperature reached was 385 °C.

The study by Bakavos and Prangnell [38], which received 205 citations, involved the use of “high power ultrasonic spot welding” (HP-USW) to weld automotive AA 6111 aluminum sheets. X-ray tomography and scanning electron microscopy were applied to analyze weld formation. From these analyses, it was observed that lower welding energies exhibited a higher defect density, while a welding energy of 750 J resulted in a more homogeneous interface with fewer defects, improving the quality of the weld. At lower welding energies, oxides remained more concentrated and uniformly dispersed at the interface, while at higher energies, their distribution became more complex and could affect the interface in more varied ways. An EBSD analysis concluded that during the welding process, areas with distinct characteristics were formed: the interface region exhibited high deformation and a finer microstructure; a shear zone revealed the material’s response to the stresses applied during welding; and finally, a forged zone due to compressive forces resulting from heating and sonotrode penetration.

The article by Elangovan, Semeer, and Prakasan [40] addressed the temperature distribution during ultrasonic metal welding using a finite element model. It explored how ultrasonic vibrations combined with pressure can efficiently join materials without excessive heating. Simulations showed that for aluminum parts, bonding occurred at an interface temperature of 336.82 °C and a clamping force of 1600 N. Thicknesses greater than 3 mm were found to be difficult to weld. The generated model highlighted the influence of material properties, surface conditions, and process variables on the welding behavior.

Harman and Albers [42] investigated the ultrasonic bonding mechanism of gold–aluminum wires in microelectronics. Their experiments demonstrated that bonding primarily occurs through deformation rather than heating. High temperatures were not essential for the formation of strong bonds. The study also emphasized the importance of contaminant removal during the process, as impurities negatively affect weld quality, and the formation of intermetallic compounds is a common cause of failure in this type of joint.

Balle, Wagner, and Eifler [43] performed a circumscribed central composite design to determine the most efficient welding parameters. Their study used Al99.5 sheets and CF-PA66 composites with thicknesses of 1 mm and 2 mm, respectively. The optimized parameters were a force of 65 N, an amplitude of 32 µm, and an energy of 1725 J, resulting in a maximum shear strength of 24.6 MPa, with failure occurring in the aluminum. Microscopic analysis showed that the polymer matrix was displaced, exposing the carbon fibers to direct contact with the aluminum. The authors concluded that both intermolecular contact and mechanical interlocking contributed to joint strength, and no fiber damage was observed.

The article by Tolunay, Dawson, and Wang [44] investigated the heating and bonding mechanisms in the ultrasonic welding of polystyrene disks. Temperature data were obtained by embedding thermocouples into the material, and the study evaluated welding force, welding time, vibration amplitude, and power consumption. Weld strength was assessed by applying torsional and compressive loads. Surface asperities allowed the interface to reach 250 °C within the first 10 ms, while the interior heated more slowly due to viscous dissipation. Higher welding force increased the heating temperature at both the interface and within the material, resulting in greater material flow out of the interface and reducing mechanical strength. Longer welding times and higher temperatures improved weld strength; however, for welding energies above 150 J, no further strength improvement was observed, and polymer chain orientation could lead to bond weakening.

### 3.2. Ultrasonic Welding + Aluminum (UW + AL)

**Figure 7:**
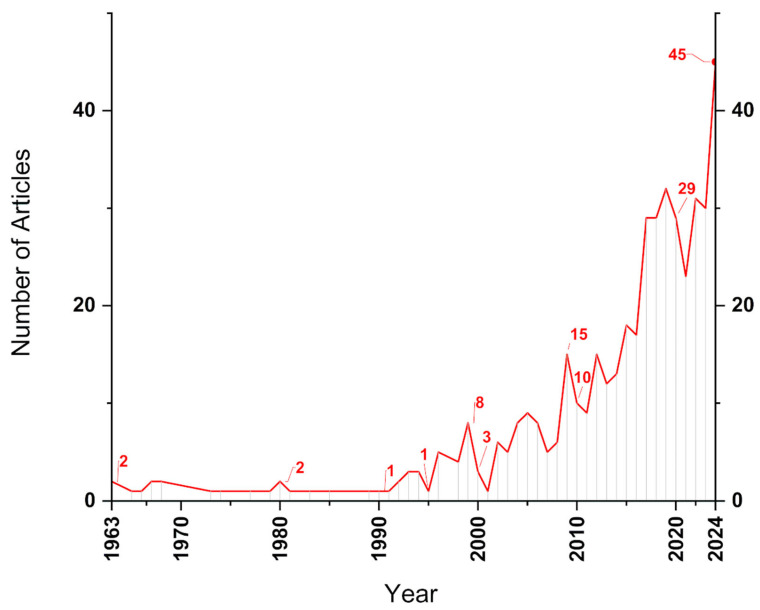
Temporal evolution of articles from the UW + Al search.

The article registry for this type of search identified a total of 454 documents, with the temporal evolution of the articles represented in Figure 7. The first articles were published in 1963, and the annual production remained below five articles until 2008, with the exception of 1998, which saw eight articles published. This period indicates progress in scientific consolidation, likely driven by improvements in infrastructure, funding, and the establishment of collaborative networks. Between 2009 and 2024, a much higher growth rate is evident compared to previous years, as noted in the previous analysis. A slight decline in article production can also be observed between 2020 and 2022, likely due to the COVID-19 pandemic. In 2023, the topic resumed its research growth, reaching the peak of the historical series with 45 articles produced in 2024, reinforcing the trend of continuous growth.

**Figure 8:**
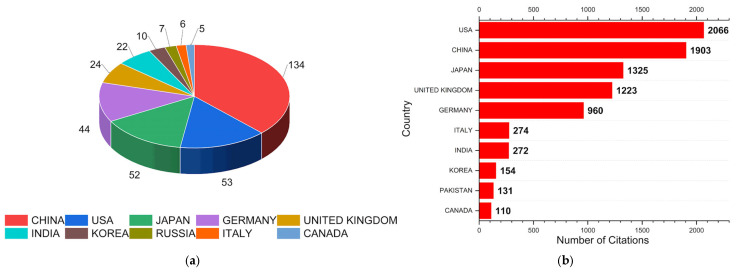
The graphs represent (**a**) the number of articles produced by country and (**b**) the number of citations obtained by country in the search for UW + Al.

According to Figure 8a, the United States leads the field with 2066 citations, reflecting its tradition in research on ultrasonic hybrid joining involving aluminum, as well as robust infrastructure for research and development in this area. As an emerging power, China occupies second place with 1903 citations, Figure 8b, revealing strong investment in this topic, which may reflect growth in the automotive and aerospace sectors. Japan also contributed significantly to the total number of citations, with 1325, demonstrating its historical relevance in materials manufacturing research. Europe, represented by the United Kingdom (1223), Germany (960), and Italy (247), shows a significant contribution, given Europe’s focus on sustainability, manufacturing technology, and materials. Following this, India, as an emerging country, has 272 citations, and this growth may be linked to the country’s focus on increasing infrastructure. Of the top 10 most cited countries, the top three account for about 63% of the total citations, demonstrating the dominance of research in this area.

The scientific production of the top 10 countries, represented in Figure 8b, shows China as the leader in production with 37.5% (134 articles). This result reflects China’s strategy of massive investment in research and technological innovation over the past few decades. In second place, the USA accounts for 14.8% (53 articles), and while not the leader, the USA maintains its historical relevance in science. Japan is also a significant player, ranking third with 14.6% (52 articles), nearly tied with the USA. These three countries together account for almost 30% of the total production. Germany, in fourth place with 12.3% (44 articles), is another major research hub, highlighting its scientific and industrial tradition. The UK and India make notable contributions with 6.7% (24) and 6.2% (22), respectively, in recent decades. Global scientific production is highly concentrated in China, the USA, Japan, and Germany. Strengthening international collaboration could foster more balanced development in this field.

Although China produces more than double the number of articles compared to the USA, it ranks second in the number of citations, trailing behind the USA. This suggests that despite its high productivity, China may have published in journals with lower impact or with less international collaboration. The USA has approximately 39 citations per article, while China has 14 citations per article. Japan maintains balance, ranking third both in the number of articles produced and citations, with an average of 25 citations per article. Italy has high relative relevance, with only six articles published but accumulating 274 citations, indicating that although its scientific production is smaller, it has a significant impact.

Table 4 presents the ten most cited articles in the search. The articles by Balle, Wagner, and Eifler [37] and Bakavos and Prangnell [38] were discussed in the previous section, so the main findings by Gunduz, Panteli, and Harman [39] will be highlighted here. The article “Enhanced diffusion and phase transformations during ultrasonic welding of zinc and aluminum” by Gunduz [39] studied the hybrid metal bonding between aluminum and zinc at 513 K. The welding interface exhibited three distinct regions: the first region with the aluminum grains of an FCC structure enriched with zinc, a second structure with a constant zinc concentration of 80 at.%, and a third structure indicating melting at the weld interface. The diffusion profile showed a thickness of approximately 3.5 µm. The study reported that zinc diffusivity in the joint was five orders of magnitude higher than the normal diffusivity for this pair at 513 K. The high deformation rates during ultrasonic welding led to a significant increase in vacancy concentration, promoting a high diffusion flux of zinc into the aluminum.

Panteli et al. [41] in “The effect of high strain rate deformation on intermetallic reaction during ultrasonic welding of aluminum to magnesium” studied the hybrid metal bonding of the AA6061 aluminum alloy and the AZ31 magnesium alloy as a function of welding energy. The authors found the presence of a structure composed of intermetallics γ-Al_12_Mg_17_ and β-Al_3_Mg_2_. The phase transformation kinetics were influenced by time and energy. Under optimal conditions (600 J and 0.4 s), the intermetallic layer thickness was 5 µm, growing to 20 µm in 1 s. Similarly to Gunduz et al. (2005) [39], the deformation rates significantly accelerated the diffusion rates compared to normal conditions. Higher welding energies led to fusion within the intermetallic layer between the magnesium substrate and the γ-Al_12_Mg_17_ sublayer. This fusion occurred at temperatures lower than the eutectic point of the Al-Mg binary system, suggesting that it was caused by localized energy dissipation during welding.

Siddiq and Ghassemieh [45] addressed the ultrasonic welding of two AA6061 aluminum sheets and the simulation integrating frictional and acoustic softening in a phenomenological model. Some key phenomena highlighted by the authors include the following: acoustic softening, provided by ultrasonic vibrations, which significantly reduces the yield stress of metals, allowing deformation under lower loads; friction, which primarily acts in removing the oxide layer at the interface, with the frictional work decreasing as the vibration amplitude increases due to acoustic softening, which reduces the resistance to sliding at the interface; and temperature control, with maximum temperatures remaining below the melting point, preserving the mechanical properties of the materials. Experimental tests and a thermomechanical analysis of the joints showed how parameters such as load and vibration affect the process, with experimental and simulated results in good agreement.

Watanabe, Sakuyama, and Yanagisawa [46] studied ultrasonic welding between SS400 carbon steel (0.8 mm thickness) and 5052 aluminum (1.2 mm thickness), using 1050 aluminum (1.2 mm thickness) as an insert. A welding frequency of 15 kHz and a power of 2400 W were applied, with clamping forces ranging from 343 N to 1764 N and welding times between 0.5 and 3 s. The optimal welding condition was found at a clamping force of 588 N and a welding time of 2.5 s. For welding times exceeding 3.0 s, the formation of Fe_2_Al_5_, a brittle intermetallic compound, was observed, reducing joint strength. The insertion of the 1050 aluminum insert tripled the joint strength, with the authors suggesting that the insert prevented the formation of brittle phases.

Matsuka and Imai [47] investigated the ultrasonic welding process of 1050 aluminum alloys (0.3 to 1.5 mm thickness) with 1020 copper (0.2 mm thickness). Ultrasonic vibrations removed oxides and surface impurities, enabling a clean interface with improved adhesion. Transition layers between Al and Cu, with thicknesses of up to 1 µm, were observed, suggesting diffusion phenomena. Although the heat input in this welding process is low, the authors proposed underwater ultrasonic welding to further minimize thermal damage to the material.

The ultrasonic welding study by Daniels [48] analyzed the ultrasonic welding of various metals, including aluminum, copper, nickel, titanium, and zirconium, examining the effects of process variables such as clamping force, ultrasonic power, and welding time. The key findings were that the minimum welding power depends on the clamping force, and weld strength increases with welding time up to a certain point (~0.8 s for aluminum). Harder materials require higher ultrasonic energy for welding. Polished surfaces enhance joint strength, while rougher surfaces reduce energy transfer. The maximum interface temperature did not exceed 40% of the metal’s melting point, suggesting that no melting occurred. Among the tested joints, the strongest was between aluminum and copper, achieved with a clamping force of 80 kg, a power of 280 W, and a welding time of 0.8 s.

Zhao, Li, and Zhang [49] investigated the failure behavior and microstructure of the weld interface between the 6061 aluminum alloy and commercially pure copper. The welding amplitude was kept constant at 12 µm, with a clamping force of 0.5 MPa, while the welding energy varied from 200 J to 2000 J, depending on the welding time. The obtained results demonstrated that joint strength increased with welding energy up to 1000 J; beyond this value, joint strength dropped drastically due to the formation of intermetallic compounds. Failure analysis revealed that for welding energies below 500 J, interfacial displacement occurred due to poor adhesion. At 1000 J, the entire joint was torn off, while at 2000 J, interfacial cleavage was observed.

### 3.3. Ultrasonic Welding + Aluminum + Composite or Polymer (UW + AL + CP)

**Figure 9:**
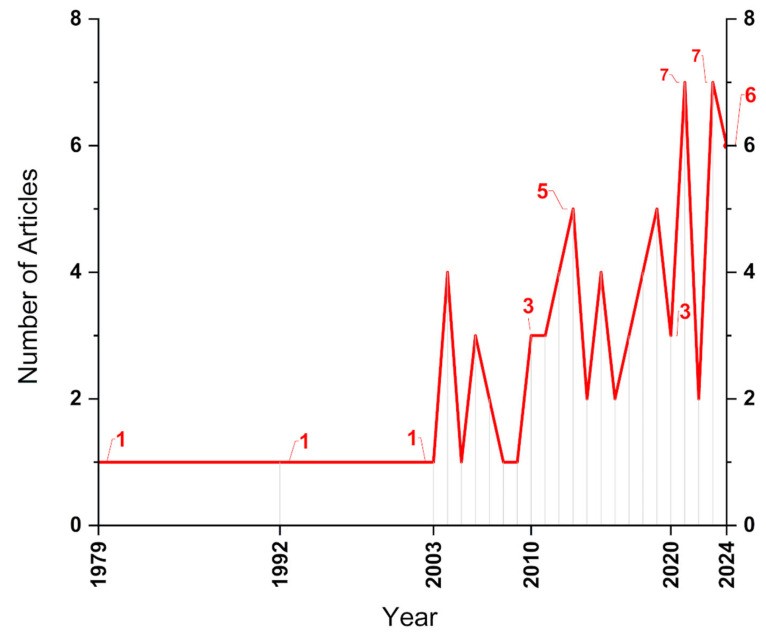
Temporal evolution of articles from the search UW + Al + CP.

Although ultrasonic welding is a relatively vast area of research, and the application of this welding technique to dissimilar materials such as aluminum and reinforced polymers reveals a significant field of opportunities, given that only 75 articles have been published on the topic. Figure 9 presents the annual evolution of scientific publications. While the first article on ultrasonic welding was recorded in 1960, the association between aluminum and polymer or composite materials was first reported in an article published in 1979. The next article was published 13 years later in 1992, and the following one was published 11 years later in 2003. In the subsequent years, there was a noticeable increase in interest in the topic, with annual productions indicating growing interest in the subject. The years with the highest number of publications were 2021 and 2023, with seven articles each, while in 2024, the total number of indexed articles in the studied databases was six. Despite the low number of published articles, the increasing interest in dissimilar ultrasonic welding suggests that research associating hybrid materials is expected to rise.

**Figure 10:**
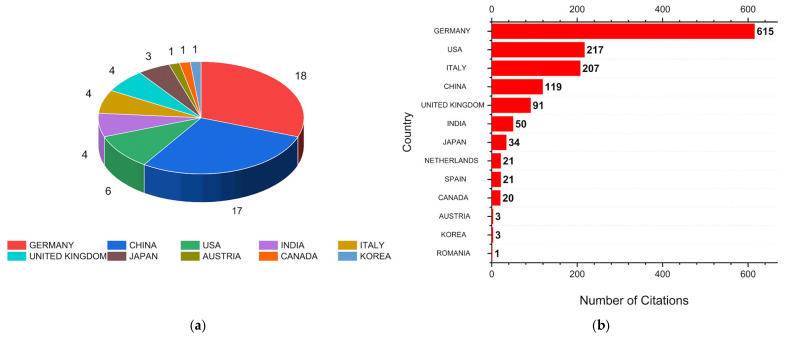
The graphs represent (**a**) the number of articles produced by country and (**b**) the number of citations obtained by country in the search for UW + Al + CP.

Figure 10a,b show the countries with the highest scientific production and citation counts, respectively. These values represent the contribution to the global scientific community. Germany, the United States, and Italy occupy the top three positions, followed by China and the United Kingdom. Each of these countries was cited 615, 217, 207, 119, and 91 times, respectively. The total citations from the remaining countries account for about 15% of the total citations, highlighting the relevance of the top five countries to the topic.

Germany stands out as the country with the highest number of publications (18) and citations (615), suggesting that the research conducted in this country is not only more abundant but also of high quality and relevance. In terms of publication volume, China (17) is close to Germany; however, the number of citations in China (119) is considerably lower, indicating that it has not yet reached the same level of impact as German studies. The U.S. has a significantly smaller number of publications compared to Germany and China but possesses a relatively high citation count (217). Even though fewer in number, American articles are influential and well received within the academic community, likely due to the reputation of the institutions and the quality of the studies conducted. India has the lowest number of publications (4) among the top five countries and a lower citation count (50), likely indicating an expansion and establishment of research foundations in the field. Finally, Italy shows a publication count (4) similar to India, but its citation count (207) is approximately four times higher, suggesting high-quality publications or articles published in journals with greater visibility.

Although the quantity of publications is not directly proportional to the impact or relevance of the research, Figure 10a,b demonstrate that countries like Germany, China, and the U.S. are at the forefront of scientific production. However, countries with lower scientific output, such as Austria and South Korea, may be focused on more specialized research areas or related topics, or may publish in journals of lower visibility in the analyzed databases. The data presented also reflect how different countries prioritize scientific research according to their institutional capabilities and scientific incentive policies.

Table 5 shows the ten most cited articles among those found. The article “Ultrasonic spot welding of aluminum sheet carbon fiber-reinforced polymer—joints” by Balle, Wagner, and Eifler [37], also applied the same experimental design as their previous studies, the central composite design (CCD), to identify the most significant parameters related to the welding process of Al99.5/CF-PA66 hybrid joints. According to the authors, the shear tensile strength of the joints exceeded 30 MPa. The welding process lasted a maximum of 5 s, without damaging the carbon fibers embedded in the polymer. The study also suggests potential applications in the automotive and aerospace industries.

Lionetto, Balle, and Maffezzoli [21] applied ultrasonic welding to AA5754 sheets and carbon fiber-reinforced epoxy. To overcome the limitation of thermoset resins at high temperatures, a polyamide 6 (PA6) film was inserted between the composite and metal, allowing for rapid welding with aluminum. By selecting the appropriate welding energy and force, an average adhesion of 34.8 MPa was achieved in the ultrasonically welded joints. Morphological evaluation revealed that the aluminum–composite interface is marked by direct contact or even the insertion of carbon fibers into the aluminum, which exhibits pores and cracks due to the pronounced plastic deformation at the aluminum interface.

In the study by Balle et al. [50], statistical experimental design principles were applied to optimize joints made from different aluminum alloys, resulting in the enhanced mechanical properties of the hybrid joints. In addition to the increased joint strength, process reproducibility was also improved through this approach. For the combination of aluminum alloy (AA1050) with carbon fiber-reinforced polyamide 66 (CF-PA66), the calculated and experimentally approved parameters resulted in the best interface properties, with failure occurring in the aluminum sheet, outside the bonding area. Further studies using scanning electron microscopy (SEM) showed a stronger and more rigid interface compared to adhesive-bonded hybrid joints, with a direct connection between the carbon fibers and the aluminum surface. Specifically, for the AA1050/CF-PA66 joints, the optimal parameters were an amplitude of 32 µm, a welding force of 60 N, and a welding energy of 1725 W, resulting in an average shear tensile strength of 24.2 ± 0.4 MPa. For the AA5754/CF-PA66 joints, a welding energy of 2160 Ws, an oscillation amplitude of 40 µm, and a welding force of 140 N provided the best welds, with the highest shear tensile strength of 32.5 ± 2.7 MPa.

Feistauer et al. [51] proposed a technique called u-joining, developed to join fiber-reinforced composite materials to metal parts manufactured by Metal Injection Molding (MIM). The study investigated the joining process between sheets with pins (four and six) of the Ti-6Al-4V alloy and GF50-PEI. The friction-generated heat softened the polymer matrix, allowing the penetration of the metal specimen’s pins, while maintaining the lamellar microstructure of Ti-6Al-4V. The joints with six and four pins exhibited approximately 5.5× and 2.4× higher shear strength compared to the pinless joint. Increasing the number of pins improved joint strength. The optimized parameter configuration for the 6-pin joint, with a welding energy of 2200 J and a pressure of 1 bar, achieved the highest mechanical strength of 2011 N.

Yang, Ram, and Stucker [52] utilized ultrasonic consolidation as a technique for the additive manufacturing of metal matrix composites reinforced with silicon carbide fibers in an aluminum 3003 laminate matrix. The results confirmed the incorporation of SiC fibers into aluminum without melting, ensuring good mechanical strength. The best strength was achieved with a 45° fiber orientation due to shear stress alignment, while a higher oscillation amplitude (20 µm) resulted in improved joint strength. This technique can be used to manufacture metal composites reinforced with long fibers.

Li et al. [53] published a study investigating the feasibility of integrating fiber Bragg gratings (FBGs) with nickel coatings (chemical or electrochemical) into aluminum and copper metal sheets with thicknesses ranging from 250 to 450 µm. When assessing the effect of welding on FBG integrity, it was observed that uncoated fibers were damaged due to pressure and vibration. Chemically nickel-coated fibers were partially preserved but exhibited cracks in the coating, whereas fibers with both chemical and electrochemical nickel coatings showed the best performance with no damage to the optical structure. This technique enabled the incorporation of FBGs into aluminum, doubling thermal sensitivity after integration.

The study by Krüger, Wagner, and Eifler [54] investigated the ultrasonic welding of metal joints (aluminum and copper) and glass fiber-reinforced PA12. The objective was to optimize welding conditions to achieve a strong and reliable metal–composite interface. The bonding occurred through a synergistic effect between mechanical interlocking and intermolecular adhesion. Surface micro-deformations allowed the penetration of the softened polymer, while the presence of fibers could hinder interfacial fusion. Mechanical strength increased up to a critical welding energy point, beyond which the polymer degraded. Higher vibration amplitudes could damage the fibers, while lower values did not provide sufficient fusion.

The study by Obielodan et al. [55] explored the ultrasonic joining of various materials (aluminum, copper, nickel, titanium, silver, molybdenum, stainless steel, and the MetPreg composite) with aluminum alloys 3003-H14 and 6061-T6. An interlayer of Al 1100 was used to facilitate the bonding of difficult-to-weld materials. Additionally, boron was introduced into the layers to modify the interface. The results showed that ultrasonic welding was viable for joining a wide range of materials, enabling different combinations. FCC materials were easier to weld to each other. The addition of boron at the titanium–aluminum interface altered the microstructure by promoting boron diffusion into aluminum. Furthermore, boron improved mechanical contact and modified the local interface properties. The authors suggested that this technique has potential applications in the aerospace and electronics industries.

### 3.4. Perspectives and Challenges

Although ultrasonic welding is well established in sectors such as automotive, electronics, and aerospace, it still presents opportunities for exploration in other areas that could increase the number of academic publications and industrial applications, namely the following:

*Medicine and Biomedicine*—Ultrasonic welding holds great potential in the production of medical devices, implants, prostheses, and biocompatible materials. These applications require a high degree of precision, ensuring that the bonding is free from contaminants and that the finish prevents the growth of harmful microorganisms. However, there is still a gap in the research concerning the mechanical behavior and biocompatibility of welded joints in biomaterials, which presents a significant opportunity for further exploration.

*Advanced Electronics and Internet of Bodies (IoB)*—This technology shows promise for miniaturized sensors in wearables devices to gather user data such as heart rate, oximetry, blood glucose, and blood pressure. Ultrasonic welding allows for the secure attachment of sensitive components, reducing the risk of harming delicate circuits. IoB devices demand very small dimensional scales, challenging the limits of ultrasonic welding technology.

*Construction and Architecture*—In the construction sector, ultrasonic welding can be valuable for joining lightweight composite panels and modular elements. While there is considerable potential for the hybrid welding of ceramics with polymers or metals, this area remains underexplored. One of the biggest hurdles is scaling the technology to work efficiently with larger components, which continues to be a key challenge for the industry.

*Nuclear Engineering*—The nuclear sector requires joints that can withstand ionizing radiation in applications such as reactors and nuclear robotics. Further studies are needed to assess the effects of radiation on the microstructure of these joints and their durability.

*Defense Sector*—In the manufacturing of unmanned vehicles, drones, and lightweight storage systems with hybrid materials, the reduction in structural weight in land, air, and sea vehicles, and the assembly of lightweight and durable weapons is critical for improving performance. Future studies should aim to explore how these hybrid joints perform under extreme mechanical, thermal, and chemical conditions.

Hybrid welding can be performed on various types of materials. For metal/polymer joints, the main engineering-reinforced polymers used were polyamide 6 (CF or GF-PA66) and polyamide 6,6 (CF or GF-PA66), likely due to their attractive mechanical, thermal, and chemical properties, as well as their affordability. Few studies use polymers such as polyether-ether-ketone (PEEK), polyphenylene sulfide (PPS), polyetherimide (PEI), polyether-ketone-ketone (PEKK), polyurethane (PU), and polysulfone (PPSU) reinforced with fiberglass or carbon fiber.

Other polymers not found in the searches include sulfonated polyetherimide (PESU), which has high chemical and thermal resistance, making it suitable for applications in harsh environments. Polyarylsulfone (PASF) offers resistance to high temperatures and good dimensional stability. Polyamide 12 (PA12) is more flexible than PA6 and PA66, with good chemical resistance, useful in applications where flexibility is a key advantage. Polyaramid (PARA) has wear resistance and excellent thermal performance, which could be explored for structural applications. Another potential field of study is the application of hybrid welding to polymers with different types of reinforcements.

## 4. Conclusions

The results obtained from the Scopus and WoS databases reveal that the scientific production on ultrasonic welding dates back to the 1960s and shows a notable growth in publications over the years. Articles on ultrasonic welding reflect a global network of collaboration, with China and the United States leading both in the number of publications and the global impact. The temporal distribution of publications indicates a significant increase in scientific output, particularly in the 2010s, with the popularization of smartphones and a growing applicability in electronic devices. Despite a slight decline in 2020 due to the COVID-19 pandemic, significant production has been recorded. The word cloud highlighted that research is increasingly focused on the use of different metals, polymers, and reinforced polymers, as well as the integration of computational methods.

Research related to ultrasonic welding and aluminum has shown that the U.S. leads in citations (2066), reinforcing its historical tradition in ultrasonic welding research. China’s high scientific output contrasts with its number of citations, suggesting that, although there are many publications, they might be in lower-impact journals or with less international collaboration. The scientific production of the ten most relevant countries is heavily concentrated in China, the U.S., Japan, and Germany, and these countries, together with international collaboration, can promote a more balanced development of the field.

Scientific production on the ultrasonic welding of aluminum and reinforced polymers began in 1979, marked by a gradual increase in interest, especially from the 2000s, with a significant peak in publications in 2021 and 2023. The citation data analysis indicates that countries like Germany, the U.S., and China are at the forefront of research, with Germany standing out for both the number of publications and the relevance of its studies, suggesting a strong quality and scientific impact. Although the ultrasonic welding of dissimilar materials, such as aluminum and polymers or composites, remains a research area with a limited number of publications—only 75 identified articles—it has proven to be a fertile ground for new opportunities and scientific growth.

Compared to traditional welding processes, ultrasonic welding offers several advantages, such as low energy consumption, high process speed, no additional consumables, low thermal input, the ability to join dissimilar metals, high precision, clean processes, and easy automation. Given these advantages, a vast window of opportunities could be explored in fields such as medicine, advanced electronics, IoB, civil engineering, architecture, nuclear energy, and the defense sector.

In summary, ultrasonic welding is an emerging field of research with high international collaboration. The continuous technical evolution, combined with the use of new methodologies and materials, promises to further drive its development, significantly contributing to the aerospace, automotive, and electronics sectors. 

## Figures and Tables

**Table 1:** Coding of keywords used in the search across databases.

Keywords	Nomenclature	Search Query Code
Ultrasonic Welding	UW	(“ultrasonic * weld *” OR “u-joining” OR “ultrasonic * join *”)
Ultrasonic Welding + Aluminum	UW + AL	(“ultrasonic * weld *” OR “u-joining” OR “ultrasonic * join *”) AND (“al” OR alumin *)
Ultrasonic Welding + Aluminum + Composite OR Polymer	UW + AL + CP	(“ultrasonic * weld *” OR “u-joining” OR “ultrasonic * join *”) AND (“al” OR alumin *) AND (“Polymer *” OR “Composite”)

**Table 2:** Distribution by journal quartiles.

Order	Source	Quartile	Number of Articles
1	Ultrasonics	Q1	38
2	Welding International	Q4	36
3	Welding Production	Not available	36
4	Journal of Manufacturing Processes	Q1	32
5	Journal of Materials Processing Technology	Q1	29
6	Materials	Q2	29
7	Automatic Welding USSR	Not available	26
8	Journal of Manufacturing Science and Engineering—Transactions of the ASME	Q1	26
9	International Journal of Advanced Manufacturing Technology	Q2	23
10	Russian Ultrasonics	Not available	23
11	Welding in the World	Q2	22
12	Welding Journal	Q2	21
13	Japanese Journal of Applied Physics	Q2	20
14	Japanese Journal of Applied Physics Part 1	Q2	20
15	Science and Technology of Welding and Joining	Q1	20
16	Composites Part A—Applied Science and Manufacturing	Q1	16
17	Journal of the Japan Welding Society	Not available	15
18	Metals	Q2	15
19	Journal of Materials Research and Technology	Q1	14

**Table 3:** Most cited articles for UW research.

No.	References	Journal	DOI	Total Citations
1	[36]	Polymer Engineering and Science	10.1002/pen.760291906	359
2	[37]	Advanced Engineering Materials	10.1002/adem.200800271	219
3	[38]	Materials Science and Engineering A	10.1016/j.msea.2010.06.038	205
4	[39]	Scripta Materialia	10.1016/j.scriptamat.2004.12.015	160
5	[40]	The International Journal of Advanced Manufacturing Technology	10.1016/j.jmatprotec.2008.03.032	160
6	[41]	Materials Science and Engineering A	10.1016/j.msea.2012.06.055	158
7	[42]	IEEE Transactions on Parts, Hybrids, and Packaging	10.1109/TPHP.1977.1135225	151
8	[43]	Materialwissenschaft und Werkstofftechnik	10.1002/mawe.200700212	147
9	[44]	Polymer Engineering and Science	10.1002/pen.760231307	145
10	[45]	Mechanics of Materials	10.1016/j.mechmat.2008.06.004	139

**Table 4:** Most cited articles for UW + Al research.

No.	References	Journal	DOI	Total Citations
1	[37]	Advanced Engineering Materials	10.1002/adem.200800271	219
2	[38]	Materials Science and Engineering A	10.1016/j.msea.2010.06.038	205
3	[39]	Scripta Materialia	10.1016/j.scriptamat.2004.12.015	160
4	[41]	Materials Science and Engineering A	10.1016/j.msea.2012.06.055	158
5	[43]	Materialwissenschaft und Werkstofftechnik	10.1002/mawe.200700212	147
6	[45]	Mechanics of Materials	10.1016/j.mechmat.2008.06.004	139
7	[46]	Journal of Materials Processing Technology	10.1016/j.jmatprotec.2009.05.006	110
8	[47]	Journal of Materials Processing Technology	10.1016/j.jmatprotec.2008.03.006	109
9	[48]	Ultrasonics	10.1016/0041-624X(65)90169-1	109
10	[49]	Science and Technology of Welding and Joining	10.1179/1362171813Y.0000000114	108

**Table 5:** Most cited articles for UW + Al +CP research.

No.	References	Journal	DOI	Total Citations
1	[37]	Advanced Engineering Materials	10.1002/adem.200800271	219
2	[43]	Materialwissenschaft und Werkstofftechnik	10.1002/mawe.200700212	147
3	[21]	Journal of Materials Processing Technology	10.1016/j.jmatprotec.2017.05.002	94
4	[50]	Materialwissenschaft und Werkstofftechnik	10.1002/mawe.201200943	70
5	[51]	Materials Letter	10.1016/j.matlet.2016.01.137	59
6	[52]	Journal of Engineering Materials and Technology	10.1115/1.2744431	58
7	[53]	Optical Fiber Technology	10.1016/j.yofte.2011.09.004	52
8	[54]	Advanced Engineering Materials	10.1002/adem.200300539	51
9	[55]	Rapid Prototyping Journal	10.1108/13552541011034843	41
10	[40]	International Journal of Advanced Manufacturing Technology	10.1007/s00170-012-3920-y	34

## Data Availability

The original contributions presented in the study are included in the article, and further inquiries can be directed to the corresponding authors.

## Supplementary Materials

The following supporting information can be downloaded at: https://www.mdpi.com/article/10.3390/polym17081124/s1, Figure S1. Temporal evolution of articles by search queries (a) UW, (b) UW + AL and (c) UW + AL + CP. Table S1. Data overview of the three search queries. Table S2. Most cited review articles of UW. Table S3. Most cited review articles of UW + AL. Table S4. Most cited review articles of UW + AL + CP. Table S5. Most relevant sources of UW. Table S6. Most relevant sources of UW + AL. Table S7. Most relevant sources of UW + AL + CP. References [16,56,57,58,59,60,61,62,63,64,65] are cited in the supplementary materials.
