# Supplementary material for: Trends and Future Projections in Ultrasonic Welding Research for Hybrid Materials
Source: Polymers (Basel). 2025 Apr 21;17(8):1124. doi: 10.3390/polym17081124 (PMC12030427; doi:10.3390/polym17081124)
Supplement: Supplementary file 1 [file polymers-17-01124-s001.zip › polymers-3429180-supplementary.pdf]

Supplementary Material

Article Title: Trends and Future Projections in Ultrasonic Welding Research for Hybrid Materials

Jedaías J. Silva [1], Rafael G. C. da Silva [2], Carolina L. Morelli [1,3], Edwar A. T. López [4] and Tiago F. A. Santos [1,3*]

[1] Brazilian Institute for Material Joining and Coating Technologies—INTM, Federal University of Pernambuco, Recife 50740-540, Brazil; jedaias.januario@ufpe.br
[2] Department of Chemical Engineering, Federal University of Pernambuco, Recife 50740-590, Brazil; rafael.casanova@ufpe.br
[3] Department of Mechanical Engineering, Federal University of Pernambuco, Recife 50740-550, Brazil; carolina.morelli@ufpe.br
[4] Department of Mechanical Engineering, Research Group GEA, University of Antioquia, Medellín 050010, Colombia; eandres.torres@udea.edu.co
* Correspondence: tiago.felipe@ufpe.br

Quantitative Analysis of Review Articles in Ultrasonic Welding

1. Introduction

This supplementary document provides an analysis of review articles related to ultrasonic welding. While these articles were excluded from the main quantitative analysis to avoid redundancy, we present here their distribution and key contributions to the field.

2. Data Collection Criteria

- The same databases (Scopus and Web of Science) were used.
- Search queries included: "Ultrasonic Welding", "Ultrasonic Welding + Aluminum", and "Ultrasonic Welding + Aluminum + Composite/Polymer".
- Only articles explicitly classified as review papers were selected.
- Duplicates were removed following the same methodology as the main analysis.

3. Data Overview

The research field has been becoming increasingly relevant over time, as observed in the annual growth and the average age of the documents in Table S1. The field is also becoming more recent and impactful. Emerging research topics are gaining popularity, which can be verified through the relationship between growth rate and the number of citations.

Table S1. Data overview of the three search queries.

**Data Type**	**UW**	**UW+AL**	**UW+AL+CP**
Timespan	1983–2025	2003 : 2025	2013 : 2023
Sources	52	24	11
Review articles	71	28	11
Annual Growth Rate of Publications	1.66%	-3.1%	7.18%
Document Average Age	7.2 years	5.43 years	4.55 years
Average Citations per Document	31.14	29.46	37.91
Average Citations per Year per Document	4.79	5.58	6.41

4. Annual Distribution of Review Articles

Figure S1 illustrates the growth in the number of review articles over the years, although a larger volume of documents is required to confirm a significant trend. However, when associating this result with the increase in scientific articles over time, as shown in Figures 3, 7, and 9, a real growth trend can be observed.

(a)

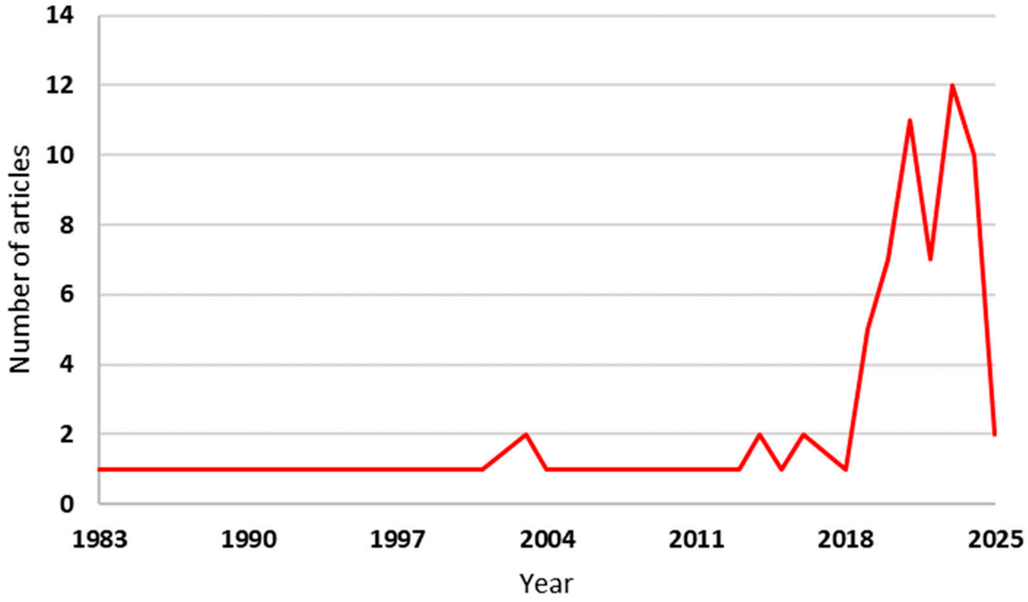

(b)

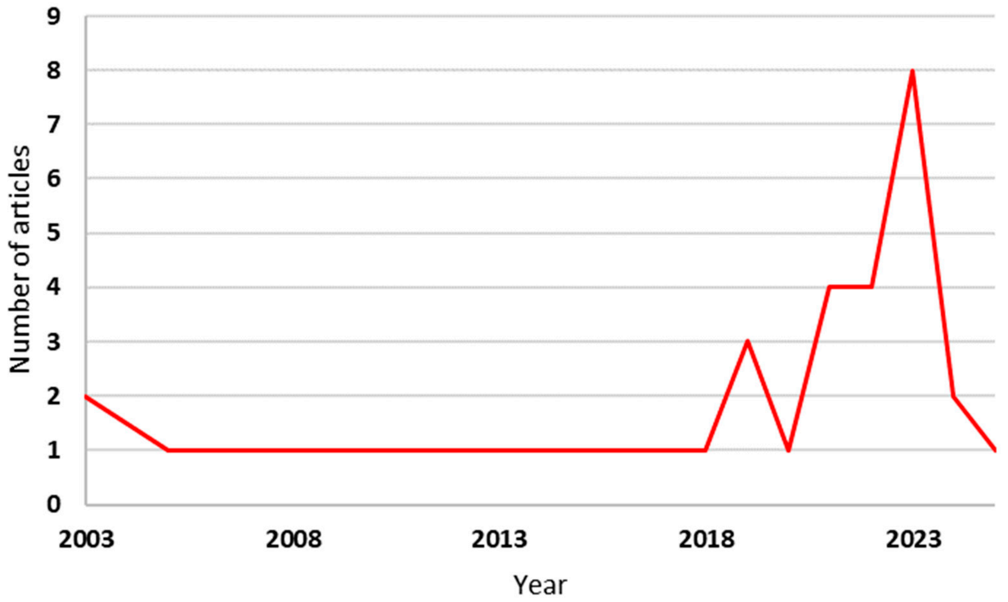

(c)

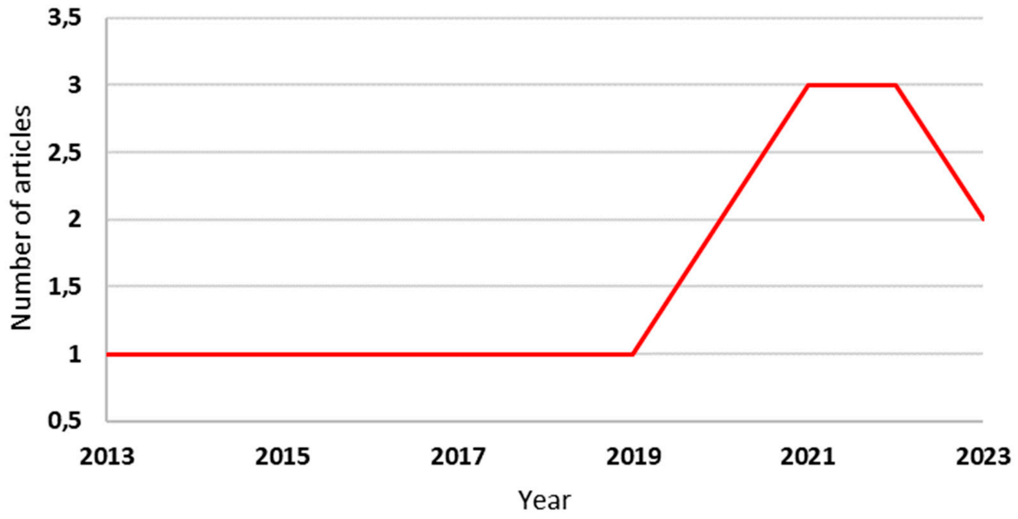

Figure S1. Temporal evolution of articles by search queries (a) UW, (b) UW + AL and (c) UW + AL + CP.

5. Top 5 Most Cited Review Articles

Tables S2, S3, and S4 present the most cited review articles for their respective search terms. The study by Ageorges C. is the most cited and the oldest, which explains its moderate citation rate. The article with the highest recent impact is Zwicker M., with the highest citation rate of 24.5 per year. Ahmed M.'s study is the most recent, with a citation rate of 22.33 per year, demonstrating rapid academic acceptance.

Table S2. Most cited review articles of UW.

Author(s)	Journal	DOI	Total Citations	Citations per Year
[1]	Composites Part A - Applied Science	10.1016/S1359-835X(00)00166-4	368	14.72
[2]	Journal of Advanced Joining Processes	10.1016/j.jajp.2020.100017	147	24.50
[3]	Journal of Manufacturing Science and Engineering - ASME	10.1115/1.4041182	133	19.00
[4]	International Polymer Processing	10.3139/217.0051	123	6.47
[5]	Materials	10.3390/ma13061284	117	19.50

Table S3. Most cited review articles of UW + AL.

Author(s)	Journal	DOI	Total Citations	Citations per Year
[3]	Journal of Manufacturing Science and Engineering - ASME	10.1115/1.4041182	133	19.00
[6]	Composites Part B-Engineering	10.1016/j.compositesb.2017.10.039	100	12.50
[7]	Advanced Engineering Materials	10.1002/adem.201300043	81	6.23
[8]	Journal of Magnesium and Alloys	10.1016/j.jma.2023.09.039	67	22.33
[9]	Polymer Engineering & Science	10.1002/pen.25022	50	7.14

Table S4. Most cited review articles of UW + AL + CP.

Author(s)	Journal	DOI	Total Citations	Citations per Year
[6]	Composites Part B-Engineering	10.1016/j.compositesb.2017.10.039	100	12.50
[7]	Advanced Engineering Materials	10.1002/adem.201300043	81	6.23
[9]	Polymer Engineering & Science	10.1002/pen.25022	50	7.14
[10]	Composite Structures	10.1016/j.compstruct.2021.113828	50	10.00
[11]	International Journal of Advanced Manufacturing Technology	10.1007/s00170-022-08753-9	39	9.75

6. Most Relevant Journals for Review Articles

Tables S5, S6, and S7 present the frequency of journals. The most relevant journals are the International Journal of Advanced Manufacturing Technology and Metals, each appearing six times, indicating their role as central sources for research on ultrasonic welding. This is followed by Advanced Engineering Materials, which appears five times. The absence of a journal specifically focused on polymers highlights a gap in the field and reinforces the pioneering nature of research in this area.

Table S5. Most relevant sources of UW.

Journal Name	Articles
CAILIAO DAOBAO/MATERIALS REPORTS	4
INTERNATIONAL JOURNAL OF ADVANCED MANUFACTURING TECHNOLOGY	3
METALS	3
ADVANCED ENGINEERING MATERIALS	2
COMPOSITES PART A-APPLIED SCIENCE AND MANUFACTURING	2

Table S6. Most relevant sources of UW + AL.

Journal Name	Articles
METALS	3
ADVANCED ENGINEERING MATERIALS	2
INTERNATIONAL JOURNAL OF ADVANCED MANUFACTURING TECHNOLOGY	2
ASSEMBLY	1
COMPOSITE STRUCTURES	1

Table S7. Most relevant sources of UW + AL + CP.

Journal Name	Articles
ADVANCED ENGINEERING MATERIALS	1
COMPOSITE STRUCTURES	1
COMPOSITES PART B-ENGINEERING	1
FRONTIERS IN MATERIALS	1
INTERNATIONAL JOURNAL OF ADVANCED MANUFACTURING TECHNOLOGY	1

5. Conclusion

The analysis of review articles enabled a quantitative investigation of another aspect of the sources on ultrasonic welding. Review articles are typically excluded from scientometric studies to avoid redundancy, as they cite original research articles. However, expanding the analysis to include reviews enhances the study, making it more aligned with bibliometric analysis.

The field of ultrasonic welding research has become increasingly relevant, as evidenced by the average annual growth and the mean age of the analyzed documents. Although a larger volume of review articles is required to confirm significant trends, the patterns identified in this study, when associated with Figures 3, 7, and 9, reinforce the existence of a growing interest in the topic.

The work by Ageorges *et al.* [1] is the most cited overall, while the article by Zwicker *et al.* [2] demonstrates the highest recent impact. Additionally, the study by Ahmed M *et al.* [8] exhibits the most notable recent growth.

The most relevant journals in this field are the International Journal of Advanced Manufacturing Technology, Metals, and Advanced Engineering Materials. The absence of a journal specifically focused on polymers highlights a gap in the literature and underscores the innovative nature of research involving this process and reinforced polymers.

References

1. Ageorges, C.; Ye, L.; Hou, M. Advances in fusion bonding techniques for joining thermoplastic matrix composites: a review. Compos. Part A Appl. Sci. Manuf. 2001, 32, 839–857.
2. Zwicker, M.F.R.; Moghadam, M.; Zhang, W.; Nielsen, C.V. Automotive battery pack manufacturing – a review of battery to tab joining. J. Adv. Join. Process. 2020, 1, 100017.
3. Cai, W.; et al. A State-of-the-Art Review on Solid-State Metal Joining. J. Manuf. Sci. Eng. 2019, 141, 031012.
4. Grewell, D.; Benatar, A. Welding of Plastics: Fundamentals and New Developments. Int. Polym. Process. 2007, 22, 43–60.
5. Bhudolia, S.K.; Gohel, G.; Leong, K.F.; Islam, A. Advances in Ultrasonic Welding of Thermoplastic Composites: A Review. Materials 2020, 13, 1284.
6. Kumar, R.; Singh, R.; Ahuja, I.P.S.; Penna, R.; Feo, L. Weldability of thermoplastic materials for friction stir welding—A state of art review and future applications. Compos. Part B Eng. 2018, 137, 1–15.
7. Wagner, G.; Balle, F.; Eifler, D. Ultrasonic Welding of Aluminum Alloys to Fiber Reinforced Polymers. Adv. Eng. Mater. 2013, 15, 792–803.
8. Ahmed, M.M.Z.; Seleman, M.M.E.; Fydrych, D.; Çam, G. Review on friction stir welding of dissimilar magnesium and aluminum alloys: Scientometric analysis and strategies for achieving high-quality joints. J. Magnes. Alloys 2023, 11, 4082–4127.
9. Feistauer, E.E.; Dos Santos, J.F.; Amancio-Filho, S.T. A review on direct assembly of through-the-thickness reinforced metal–polymer composite hybrid structures. Polym. Eng. Sci. 2019, 59, 661–674.
10. Lambiase, F.; Balle, F.; Blaga, L.-A.; Liu, F.; Amancio-Filho, S.T. Friction-based processes for hybrid multi-material joining. Compos. Struct. 2021, 266, 113828.
11. Li, H.; Chen, C.; Yi, R.; Li, Y.; Wu, J. Ultrasonic welding of fiber-reinforced thermoplastic composites: a review. Int. J. Adv. Manuf. Technol. 2022, 120, 29–57.
